# Anti-Cancer Activity of a 5-Aminopyrazole Derivative Lead Compound (BC-7) and Potential Synergistic Cytotoxicity with Cisplatin against Human Cervical Cancer Cells

**DOI:** 10.3390/ijms20225559

**Published:** 2019-11-07

**Authors:** Bresler Swanepoel, George Mihai Nitulescu, Octavian Tudorel Olaru, Luanne Venables, Maryna van de Venter

**Affiliations:** 1 Department of Biochemistry and Microbiology, Nelson Mandela University, P.O. Box 77000, Port Elizabeth 6031, South Africa; s211129399@mandela.ac.za (B.S.); s204004039@mandela.ac.za (L.V.); Maryna.VanDeVenter@mandela.ac.za (M.v.d.V.); 2 Faculty of Pharmacy, “Carol Davila” University of Medicine and Pharmacy, Traian Vuia 6, 020956 Bucharest, Romania; octavian.olaru@umfcd.ro

**Keywords:** 5-aminopyrazole derivative, cytotoxicity, apoptosis, cisplatin, combination therapy, synergism, cervical cancer

## Abstract

The use of some very well-known chemotherapeutic agents, such as cisplatin, is limited by toxicity in normal tissues and the development of drug resistance. In order to address drug resistance and the side-effects of anti-cancer agents, recent research has focused on finding novel combinations of anti-cancer agents with non-overlapping mechanisms of action. The cytotoxic effect of the synthetic 5-aminopyrazole derivative *N*-[[3-(4-bromophenyl)-1*H*-pyrazol-5-yl]-carbamothioyl]-4-chloro-benzamide (BC-7) was evaluated by the bis-Benzamide H 33342 trihydrochloride/propidium iodide (Hoechst 33342/PI) dual staining method against HeLa, MeWo, HepG2, Vero, and MRHF cell lines. Quantitative fluorescence image analysis was used for the elucidation of mechanism of action and synergism with cisplatin in HeLa cells. BC-7 displayed selective cytotoxicity towards HeLa cells (IC_50_ 65.58 ± 8.40 μM) and induced apoptosis in a mitochondrial- and caspase dependent manner. This was most likely preceded by cell cycle arrest in the early M phase and the onset of mitotic catastrophe. BC-7 increased the cytotoxic effect of cisplatin in a synergistic manner with combination index (CI) values less than 0.9 accompanied by highly favourable dose reduction indices. Therefore, the results obtained support the implication that BC-7 has potential anti-cancer properties and that combinations of BC-7 with cisplatin should be further investigated for potential clinical applications.

## 1. Introduction

The low rates of survival for most metastatic cancers, within a 5-year period, coupled with the lengthy and sometimes costly process of developing new anti-cancer drugs has led to the consideration of new treatment strategies [1,2]. Such strategies include that of combination therapy which can be defined as a treatment modality that combines two or more therapeutic agents in order to primarily reduce the dose of treatment that in turn would aid in reduced drug resistance as well as toxicity towards normal host cells. This approach mainly involves the repurposing of a known Food and Drug Administration (FDA)-approved agent, though it is not necessarily always used for the treatment of cancer, and combining it with another drug in order to reduce overall costs, ultimately benefiting patients in developing countries [2].

Cervical cancer is the second most common type of cancer diagnosed in women worldwide. It is one of the leading contributors to death in young females and the development thereof occurs in the cells that constitute the lining of the cervix [3]. Approximately 85% of cervical cancers are characterised as squamous cell carcinomas, which vary in growth pattern and morphology. Cisplatin monotherapy has been deemed to have the best response and survival rates. It was reported that upon treatment with cisplatin, for three weeks, the overall response rate was between 20%–30% and the survival rate for patients was approximately seven months [4].

Cisplatin is a platinum derivative that was initially approved by the FDA in 1978 for the treatment of testicular cancer [5]. M. Peyrone was the first to synthesise cisplatin in 1844 and Alfred Werner was the first to characterise its chemical structure in 1893. Scientific investigations of cisplatin only started in the 1960s after it was shown to inhibit the cell division of *E. coli* [6]. Since then, cisplatin was shown to have anti-cancer activity against a variety of cancers and remains one of the most widely used chemotherapeutic agents to date. However, increasing evidence associated with cisplatin treatment indicates drug resistance by cancer cells and considerable side effects in patients. These side effects include nausea, vomiting, myelosuppression (decreased blood cell and platelet production in bone marrow), immunosuppression, nephrotoxicity, hepatotoxicity, and cardiotoxicity [7,8]. All these factors ultimately limit the dose of cisplatin that can be administered. Therefore, research has been aimed at the combination of cisplatin with commonly used chemotherapeutic drugs and natural agents such as paclitaxel, tegafur-uracil, doxorubicin, gemcitabine, 5-fluorouracil, metformin, vitamin D, bleomycin, vinblastine, methotrexate, and honeybee venom [7].

Resistance of cancer cells to chemotherapeutic agents has been said to occur in two forms, namely acquired and intrinsic resistance. Acquired resistance refers to a drug that becomes ineffective over time whereas intrinsic resistance refers to a drug that is ineffective from the onset of treatment. Cisplatin resistance is a form of acquired resistance. Mechanisms involved in cisplatin resistance include: (1) Decreased intracellular accumulation or increased efflux, (2) drug inactivation, (3) alteration of drug target, (4) increased nucleotide excision-repair activity, (5) decreased mismatch-repair activity, and (6) evasion of apoptosis [9,10,11].

*N*-[[3-(4-bromophenyl)-1*H*-pyrazol-5-yl]-carbamothioyl]-4-chloro-benzamide (BC-7) is a synthetic 5-aminopyrazole thiourea derivative that has been developed through rapid ultrasound mediated methods as described by Nitulescu et al. [12]. An extensive review by Karrouchi et al. [13] details several pyrazole derivatives that have been shown to have various biological properties such as antimicrobial, anticonvulsant, analgesic, anti-inflammatory, anti-viral, anti-malarial, and anti-cancer. Due to these properties, researchers at present are focused on designing derivatives with greater biological diversity [13]. The anti-cancer activity and apoptosis inducing effect of BC-7 has been outlined using HT-29 human colorectal adenocarcinoma cells using cell cycle analysis, annexin-V/FITC staining, and real-time PCR. Cell cycle analysis results obtained by Nitulescu et al. [12] indicated that BC-7 induces G2/M phase arrest. Annexin-V/FITC staining performed by Nitulescu et al. [12] indicated a marked increase in the amount of early apoptotic and necrotic cells with the most significant increase seen in the latter. Relative gene expression for pro-apoptotic genes such as Bcl-2, MCL1, and caspase 8 and 3 were also shown to have increased compared to the untreated control [12].

Although the anti-cancer potential of BC-7 has previously been reported, this study set out to elaborate on the mechanism of action of cell death and to provide evidence of synergistic cytotoxic activity when combined with cisplatin.

## 2. Results

### 2.1. Cytotoxicity

The cytotoxic effect of BC-7 and cisplatin against HeLa cervical cancer cells was assessed after 48 h of treatment. The Hoechst 33342/PI dual staining method was used and IC_50_ values obtained were 65.58 ± 8.40 μM and 1.675 ± 0.301 μM, respectively. Treatment of MeWo skin melanoma and HepG2 hepatoblastoma cells with BC-7 and cisplatin showed that BC-7 was not cytotoxic to these cell lines with IC_50_ values of >200 μM. Cisplatin treatment yielded IC_50_ values of 9.300 ± 1.527 μM and 3.525 ± 1.521 μM against MeWo and HepG2 cells, respectively. BC-7 treatment of immortalised and non-tumorigenic cells, namely Vero African Green Monkey Kidney and MRHF human foreskin fibroblasts led to IC_50_ values of 169.5 ± 27.45 μM and 111.4 ± 9.300 μM being obtained, respectively. Likewise, cisplatin treatment yielded IC_50_ values of 5.921 ± 0.810 μM and 6.490 ± 1.782 μM, respectively. The IC_10_, IC_25_, and IC_75_ values for HeLa cells were determined (Table 1) and, together with the IC_50_ values, were used for all the subsequent experiments. The cytotoxic effect of the aforementioned concentrations (Table 1), after 24 h of exposure, was assessed to illustrate the possible time-dependence of BC-7 treatment. Cell death percentages of 6.906 ± 0.132%, 6.418 ± 3.331%, 8.822 ± 1.189%, and 32.79 ± 4.604% were calculated, respectively, indicating that treatment with BC-7 was indeed time-dependent as these concentrations led to 10%, 25%, 50%, and 75% cell death after 48 h of exposure.

The selectivity index (SI) of BC-7 and cisplatin for the cancer cells were calculated relative to the normal cells by dividing the IC_50_ value of the normal cells by that of the cancer cells (Table 2). A SI value of less than 2 indicates general toxicity [14]. When comparing HeLa cell toxicity to that of Vero cell toxicity, cisplatin and BC-7 indicated SI values of 3.53 and 2.58, respectively. Compared to MRHF cells, only cisplatin displayed any significant selectivity with a SI value of 3.87. Both cisplatin and BC-7 showed to have greater selectivity toward HeLa cells compared to MeWo and HepG2 cells.

### 2.2. Cell Cycle Analysis

**Figure 1:**
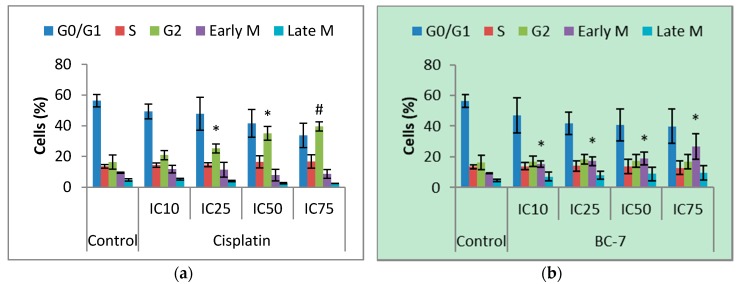
Cell cycle analysis of HeLa cells after 24 h treatment with (**a**) cisplatin and (**b**) BC-7. Cell cycle analysis was determined by the NucRed^™^ Live 647 staining method. Control treatment refers to untreated control. Results displayed as percentage of cells detected in each phase. Error bars indicate SD of three individual experiments, each performed in quadruplicate (*n* = 3). Significance was determined using the two-tailed student t-test: * *p* < 0.05 and ^#^ *p* < 0.005 compared to untreated control.

Cell cycle phase distribution was evaluated to further characterise the cytotoxic effect of BC-7 and cisplatin on HeLa cells. Significant dose dependent, phase arrest after 24 h was observed in the early M phase for the BC-7 treatment (*p* < 0.05) and in the G2 phase for the cisplatin treatment, respectively (Figure 1). The same observations were made after 48 h. However, a significant increase in the number of cells arrested in the G2 phase was also evident for treatment with BC-7 (Figure S1).

### 2.3. Histone H3 Phosphorylation

Entry into mitosis for the BC-7 treatment was confirmed by immunofluorescence staining with phospho-H3 (Ser10) rabbit mAb after 24 and 48 h (Table 3). Cisplatin treatments indicated a dose dependent decrease in positive staining for phosphorylated histone H3 after 24 h, supporting the G2 phase arrest. However, the same trend was not seen after 48 h as the IC_50_ treatment of cisplatin indicated a significant increase. Treatment with BC-7 after 24 h, but not 48 h, indicated significant increases in positive staining. It should also be noted that there was a general decrease in positive staining after 48 h as compared to 24 h.

### 2.4. Micronuclei Formation

**Figure 2:**
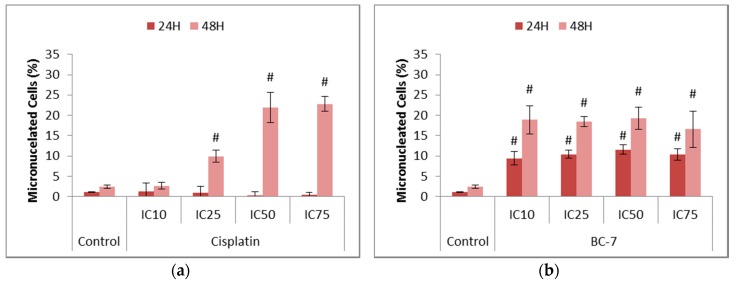
Assessment of micronuclei formation in HeLa cells after 24 and 48 h treatment with (**a**) cisplatin and (**b**) BC-7. The NucRed™ Live 647 staining method was used. Control treatment refers to untreated control. Results displayed as percentage micronucleated cells. Error bars indicate SD of three individual experiments, each performed in quadruplicate (*n* = 3). Significance was determined using the two-tailed student *t*-test: ^#^ *p* < 0.005 compared to untreated control.

The onset of mitotic catastrophe through treatment with cisplatin and BC-7 was evaluated by the measurement of micronuclei formation (Figure 2). Treatments with BC-7 after 24 and 48 h led to significant dose dependent increases in micronuclei formation. Cisplatin on the other hand showed no effect on micronuclei formation after 24 h and a significant dose dependent effect after 48 h.

To explore the mechanism of BC-7 and cisplatin-induced apoptosis in HeLa cells, phosphatidylserine translocation, mitochondrial membrane depolarisation, caspase activation, Reactive Oxygen Species (ROS) production, NF-κB activation, and autophagy induction was evaluated.

### 2.5. Phosphatidylserine Translocation

**Figure 3:**
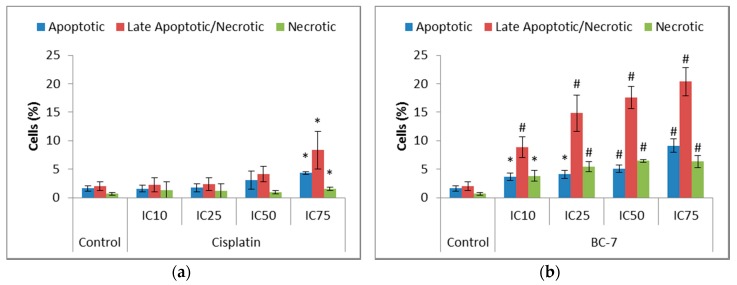
Analysis of phosphatidylserine (PS) translocation in HeLa cells after 48 h of treatment with (**a**) cisplatin and (**b**) BC-7. The Annexin V-FITC/PI dual staining method was used. Control treatment refers to untreated control. Results displayed as percentage positively stained cells. Error bars indicate SD of three individual experiments, each performed in quadruplicate (*n* = 3). Significance was determined using the two-tailed student t-test: * *p* < 0.05 and ^#^ *p* < 0.005 compared to untreated control.

The induction of apoptosis leads to the loss of membrane assymetry, subsequently causing the translocation of phosphatidylserine (PS) from the inner to the outer leaflet of the cell membrane. Greater significant increases were seen for all BC-7 treatments, in a dose dependent manner, after 48 rather than 24 h, compared to the control as opposed to treatment with cisplatin (Figure 3).

### 2.6. Mitochondrial Membrane Potential (MMP) Analysis

Mitochondrial membrane depolarisation is a marker for the onset of the intrinsic mode of apoptosis and the subsequent release of cyt-c. All treatments indicated a dose dependent loss in MMP after 24 and 48 h (Table 4). The most significant loss (*p* < 0.005) was seen after 48 h for treatment with both cisplatin and BC-7, respectively.

### 2.7. Caspase 8 and 3 Activation

Caspase 8 and 3 are the initiator and executioner caspases responsible for the onset of the caspase cascade and the execution of apoptosis, respectively, as observed by the morphological and biochemical changes in cells. Caspase activity (Table 5 and Table 6) indicated that caspase 8 and 3 activities were elevated over the untreated control, for treatments with BC-7 and cisplatin, in a dose dependent manner.

### 2.8. Reactive Oxygen Species Production

**Figure 4:**
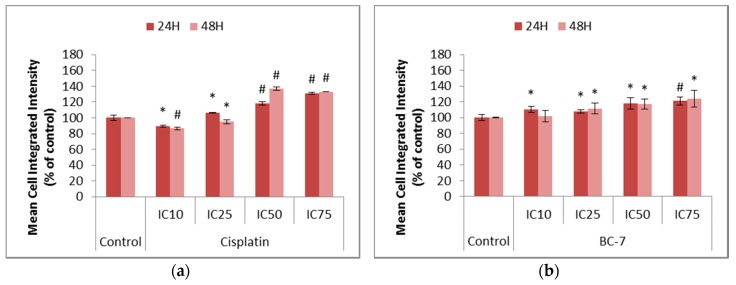
Changes in the levels of Reactive Oxygen Species (ROS) in HeLa cells after 24 and 48 h of treatment with (**a**) cisplatin and (**b**) BC-7. The CellRox^®^ Orange staining method was used. Control treatment refers to untreated control. Results displayed as mean cell integrated intensity and expressed as a percentage of the untreated control. Error bars indicate SD of three individual experiments, each performed in quadruplicate (*n* = 3). Significance was determined using the two-tailed student *t*-test: * *p* < 0.05 and ^#^ *p* < 0.005 compared to untreated control.

The production of Reactive Oxygen Species (ROS) was determined to assess its role in the induction of cell survival or cell death in HeLa cancer cells (Figure 4). Cisplatin was used as the positive control as it has been reported to promote the production of ROS. All treatments with BC-7 indicated significant increases in the amount of ROS after both 24 and 48 h in a dose dependent manner. Significant decreases in ROS after 48 h, compared to the untreated control, was seen for the IC_10_ (13.6%) and IC_25_ (5.1%) of cisplatin. The IC_10_ of cisplatin after 24 h also indicated a significant 10.9% decrease in ROS, compared to the untreated control. At higher cisplatin concentrations, the expected increase was observed at both time points.

### 2.9. Nuclear Factor kappa B Activation

**Figure 5:**
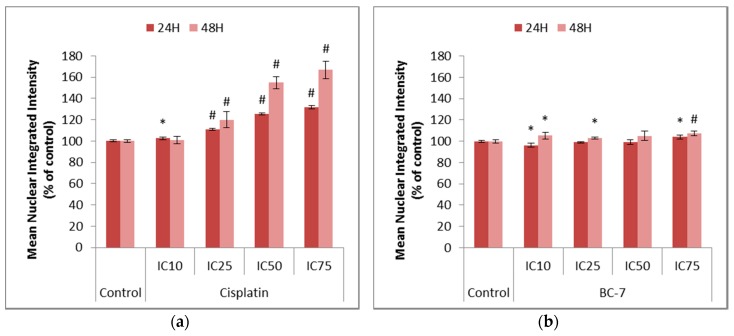
Nuclear Factor kappa B (NF-κB) analysis in HeLa cells after 24 and 48 h of treatment with (**a**) cisplatin and (**b**) BC-7. Immunofluorescence staining with phospho-p65 NF-κB was done. Control treatment refers to untreated control. Results displayed as a mean nuclear integrated intensity and expressed as a percentage of the untreated control. Error bars indicate SD of three individual experiments, each performed in quadruplicate (*n* = 3). Significance was determined using the two-tailed student *t*-test: * *p* < 0.05 and ^#^ *p* < 0.005 compared to untreated control.

Nuclear Factor kappa B (NF-κB) is constitutively expressed in most types of cancers and causes the activation of anti-apoptotic factors such as cellular inhibitors of apoptosis and cell cycle progression factors such as cyclins. NF-κB mediates the transcription of various target genes such as iNOS and COX-2, which are responsible for the formation of nitric oxide (NO) and prostaglandins such as PGE2, respectively. Ultimately, these products interfere with cellular processes that may result in the development and progression of cancer [15]. Mangiferin was used as a positive control as it is a natural polyphenol with known anti-inflammatory, anti-oxidant, and anti-viral properties. Treatment with mangiferin induced an 18.1% decrease in NF-κB translocation after 48 h as compared to the untreated control (Figure S2). All treatments with cisplatin led to significant increases in NF-κB activation (Figure 5). Likewise, most treatments with BC-7 also indicated small, but significant, increases in NF-κB activation except for the IC_10_ treatment which indicated a slight significant decrease by 3.8% after 24 h.

### 2.10. Induction of Autophagy-Related Processes

**Figure 6:**
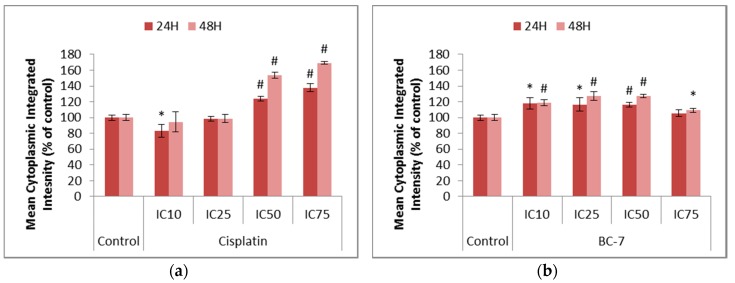
Changes in the levels of acidic organelles in HeLa cells after 24 and 48 h of treatment with (**a**) cisplatin and (**b**) BC-7. The LysoTracker™ Deep Red staining method was used. Control treatment refers to untreated control. Results displayed as mean cytoplasmic integrated intensity and expressed as a percentage of the untreated control. Error bars indicate SD of three individual experiments, each performed in quadruplicate (*n* = 3). Significance was determined using the two-tailed student *t*-test: * *p* < 0.05 and ^#^ *p* < 0.005 compared to untreated control.

The induction of autophagy-related processes was indirectly assessed using LysoTracker™ Deep Red staining, which stains acidic organelles such as late autophagic vesicles, to identify whether BC-7 and cisplatin had the potential to promote cell death in a non-apoptotic manner. An induction of autophagy is also believed to play a role in cell survival and drug resistance. Chloroquine served as a positive control and significantly (*p* < 0.005) increased the number of acidic organelles after 24 and 48 h of treatment (Figure S3). Cisplatin and BC-7 treatments led to significant increases in the mean cytoplasmic integrated intensity correlating to an increase in the number of acidic organelles. (Figure 6).

### 2.11. Combination Therapy

**Figure 7:**
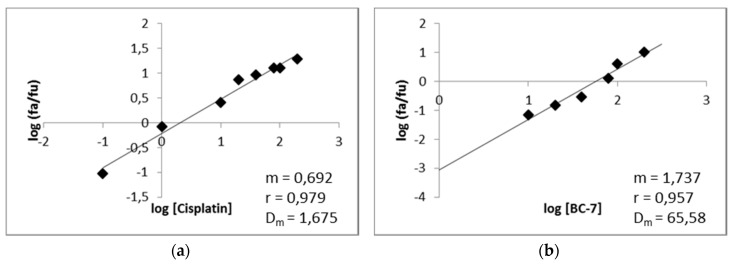
Median-effect plots of (**a**) cisplatin and (**b**) BC-7 for HeLa cells. After 48 h of exposure, the cell death percentage was determined with the Hoechst 33342/PI assay to determine the *m* value (a measurement of the shape or sigmoidity of the dose-effect) and the *r* value (a linear correlation coefficient of the median-effect plot).

Median-effect plots for cisplatin and BC-7 were generated from dose response curves (Figure 7). These plots were generated by plotting the log of the fraction of cells affected (fa) over the fraction of cells unaffected (fu) versus the log of the dose, for each of the compounds. The shape (m), potency (Dm), and conformity (*r*) of the data to the mass action law for each of the compounds are also displayed.

The potential for synergistic cytotoxic activity between BC-7 and cisplatin was evaluated using the CI theorem along with the median effect plot parameters in Figure 7. The combination index (CI) and dose reduction index (DRI) values at 10%, 25%, 50%, and 75% inhibition of HeLa cell growth, for each of the combination ratios, were calculated based on Tables 10 and 11 of Chou [16]. Based on the CI values most of the combinations at the different ratios (as described in Section 2.8), for all the effect levels indicated synergism (CI < 0.9) to varying degrees. Exceptions could be made for the 1:2 ratio at 50% inhibition and the 1:3 and 1:2 ratios at the inhibition of 75%. Favourable dose reduction (DRI > 1) was observed for all combinations for at least one of the compounds, except for the 1:3 and 1:2 ratios at the inhibition of 75% of HeLa cell growth, which indicated unfavourable dose reduction for BC-7.

**Figure 8:**
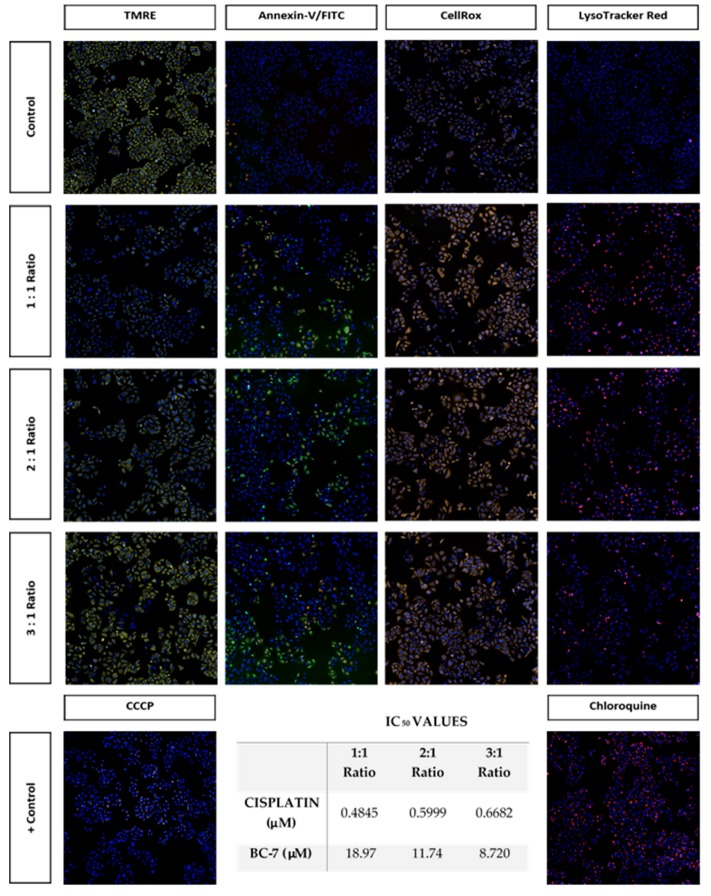
Photomicrographs (10× magnification) illustrating the effects of synergistic combinations of cisplatin and BC-7 at reduced IC_50_ values on HeLa cells after 48 h of treatment. All three combination treatments significantly reduced the average number of cells per site observed relative to the control by approximately 50% (Figure S4). There was also a significant increase in the nuclei mean area relative to the control (Figure S5).

The effects of the 1:1, 2:1, and 3:1 combinations, at the inhibition of 50% of HeLa cell growth, which were determined to be synergistic having CI values of 0.53, 0.54, and 0.58 were assessed using the reduced IC_50_ doses of cisplatin and BC-7 for each combination, respectively. Photomicrographs (Figure 8) illustrate that these combinations were indeed able to induce a response in HeLa cells at these reduced doses, confirming that these combinations are synergistic.

## 3. Discussion

BC-7 is a 5-aminopyrazole derivative that was synthesised as a cell cycle kinase inhibitor based on the prominent pyrazole ring that is used extensively in the design of compounds targeted to block cell cycle progression [12]. BC-7 has been reported to be cytotoxic against HT29 and THP1 cells and the mechanism of action has been outlined using HT29 cells [12]. HeLa cells were used in this study to further describe the mechanism of action by focusing on additional parameters of cell death induction. Dose response assays showed that the IC_50_ value of 65.58 ± 8.400 μM for treatment of HeLa cells with BC-7 was 1.4 times lower than that reported on HT29 cells. The cytotoxic effect of BC-7 on control cell lines such as Vero and MRHF cells has never been reported. This study was therefore the first to report that BC-7 yielded IC_50_ values of 169.5 ± 27.45 μM and 111.4 ± 9.300 μM on Vero and MRHF cells, respectively. It should however be noted that cells were seeded at a density considered to be representative of proliferating cells rather than a confluent monolayer. The lower IC_50_ on proliferating cells therefore pointed towards a cell cycle dependent mechanism of action. BC-7 treatment also showed to have no effect on HepG2 and MeWo cells, supporting the fact that cancer cells accumulate multiple mutations that affect their sensitivity toward anti-cancer treatment [17]. HepG2 cells are known to have a low expression of phosphatase and tensin homologue (PTEN) whereas MeWo cells are characterised as a p53 mutant, respectively. These mutations may explain why HeLa cells were more susceptible to treatments as supported by the greater SI values (Table 2), compared to MeWo and HepG2 cells. However, these are merely examples of well-known mutations within these cell lines and it should be noted that many more exist.

Disruption of the cell cycle due to DNA damage, leading to the inhibition of cell proliferation, is a key characteristic of many chemotherapeutic agents such as cisplatin, 5-fluorouracil, and paclitaxel. Treatment with cisplatin caused G2 phase arrest (Figure 1) which is supported by numerous studies that have reported a G2/M phase arrest [18,19,20]. Likewise, the study by Nitulescu et al. [12] also reported a G2/M phase arrest for a treatment with BC-7. These previous studies made use of flow cytometry for cell cycle analysis: A method that cannot distinguish between cells in G2 and M phase without additional phase specific markers such as histone H3 phosphorylation. The image-based method employed in the current study overcame this limitation and confirmed an early M phase arrest for BC-7. However, detection of histone H3 phosphorylation was included in this study (Table 3) and showed that treatment with BC-7, after 24 h, did indeed induce phosphorylation, supporting the early M phase arrest. This was, however, not the case after 48 h but there was a noticeable increase in cells arrested at the G2 phase. Treatment with cisplatin led to a dose dependent decrease in histone H3 phosphorylation after 24 h with hardly any effect seen after 48 h. Therefore, supporting the G2 phase arrest observed.

All treatments with BC-7 indicated a significant increase in the formation of micronucleated cells which, in addition to the observed early M phase arrest, suggests the possible onset of mitotic catastrophe (Figure 2). This observation is supported by results obtained from Cui et al. [21] and Mrkvova et al. [22] which have shown that a series of 1*H*-benzofuro[3,2-c]pyrazole derivatives and benzimidazoles affect microtubule formation, respectively. Taken together with the cell cycle analysis results, it could be said that cisplatin does not induce mitotic catastrophe as cells were arrested in the G2 phase. However, research has shown that, upon treatment with genotoxic agents such as cisplatin, some cancer cells can overcome a G2 phase arrest and enter mitosis. This is a process known as G2/M checkpoint adaptation [23]. It was also found that G2/M checkpoint adaptation led to the formation of micronuclei. Cells containing micronuclei were also found to be capable of surviving several cycles and the chromosomes enclosed in the micronuclei can reunite with the main nucleus.

A study completed by Lewis and Golsteyn [24] confirmed that, upon treatment of glioblastoma cells with cisplatin, G2/M checkpoint adaptation occurred and was followed by an increase in the formation of micronuclei. This could possibly explain why there was an indication of micronuclei formation after 48 h of treatment with cisplatin, but not after 24 h of treatment. However, there was no significant increase in the percentage of cells arrested in mitosis after 48 h. Thus, suggesting that the formation of micronuclei may occur elsewhere. Interestingly, it has been found that micronuclei can also be formed during interphase. This is said to occur due to a process known as nuclear blebbing in which extrachromosomal pieces of the main nucleus are transported to the nuclear envelope, forming a bud which when separated forms a micronucleus [23]. Therefore, cisplatin could induce micronuclei formation in a different manner compared to BC-7.

Changes in the cellular membrane are characteristic of apoptosis, thus such modifications can be used to identify and distinguish apoptotic cells from the rest of the population. One of these modifications, which can be detected by Annexin V-FITC/PI staining, is the ability of PS to translocate to the outer leaflet of the cell membrane during apoptosis [25]. The results obtained for treatment with BC-7 indicated a significant dose dependent increase in the number of cells staining positive for Annexin V-FITC and/or PI after 48 h (Figure 3). BC-7 has previously been shown to induce PS translocation after 24 h in HT29 cells with 5.69% of cells being categorised as apoptotic, 1.16% as late apoptotic/ necrotic, and 8.43% as necrotic [12]. Treatment with cisplatin did not lead to a significant increase in positive staining for Annexin V-FITC and/or PI after 48 h, except at its IC_75_. However, cisplatin treatment indicated small but significant increases after 24 h. Thus, suggesting that the induction of apoptosis by cisplatin occurs faster compared to BC-7.

Disruption of the mitochondrial membrane potential and the activation of the caspase proteolytic cascade are perhaps the main features of apoptosis induction. This is mainly because these features play an overwhelming role in the two main pathways in which apoptosis can be induced. Treatment with BC-7 for 24 and 48 h indicated significant dose dependent decreases in the mean cytoplasmic integrated intensity of TMRE (Table 4) and therefore suggests the involvement of the mitochondria in the onset of apoptosis.

Increases in the mean cell integrated intensities of both caspase 8 and 3 after 24 and 48 h, respectively, was evident in a dose dependent manner for all treatments to varying degrees (Table 5 and Table 6). Treatment with BC-7 led to significant increases in caspase 8 and 3 activation after 48 h but not 24 h and could suggest the involvement of both the extrinsic and intrinsic pathway in the execution of apoptosis. However, it seems that an activation of the caspase cascade was slower compared to cisplatin treatment. This is also supported by the results obtained for the mitochondrial membrane potential as there was no significant decrease after 24 h, and PS translocation as there was more PS present after 48 h.

Depending on the levels, ROS can function as either a cell death or cell survival mediator [26]. Cancer cells are known to have an increased level of ROS compared to normal cells. However, it is when these levels are exacerbated, normally under hypoxic conditions, that ROS can lead to DNA damage and an activation of p53 signalling ultimately resulting in genetic stress and instability [27,28,29]. Most treatments, with a few exceptions at the lowest concentrations of cisplatin, led to a significant increase in ROS levels (Figure 4). ROS does not act through the extrinsic pathway of apoptosis but can influence the intracellular environment in order to enhance receptor-ligand engagement or the execution of the downstream events leading to apoptosis. The up-regulation of CD95 and TRAIL death receptors has already been observed in response to hydrogen peroxide. Cisplatin served as the positive control as it is known to induce CD95 clustering by increasing intracellular ROS [30] and treatment with antioxidants has shown to counteract this effect. ROS has been shown to sensitise cancer cells to TRAIL-induced apoptosis and caspase activation. Caspases are known to function optimally under reducing conditions therefore proteins that can antagonise an increase in cellular ROS levels can protect cancer cells from ROS mediated apoptosis [31].

Cisplatin resistance due to the evasion of apoptosis has been proposed to occur due to the induction of autophagy in ovarian cancer cells [32,33] as well as the activation of NF-κB in pancreatic carcinoma cells [7,20]. Significant increases for cisplatin treatment were seen in the mean cytoplasmic and nuclear integrated intensities representative of the induction of autophagy-related processes (Figure 6) and NF-κB activation (Figure 5), respectively. This supports the abovementioned statement on the cause of cisplatin resistance. Treatment with BC-7 showed to also activate NF-κB and induce autophagy-related processes, but to a lesser extent compared to cisplatin.

Combination chemotherapy with cisplatin forms the basis of cancer treatments such as ovarian, biliary tract, lung, gastric, prostate, melanoma, breast, colon, pancreatic, and cervical cancer. Although platinum responsiveness is high during the initial stages of treatment, numerous patients have relapsed with cisplatin-resistant disease [8]. Therefore, one of the most important objectives of combination therapy is the ability to reduce the dose of a drug used, thus reducing toxicity toward the host while maintaining therapeutic efficacy (Figure 8). The dose contributions of each drug in combination can be determined and a DRI can be calculated, relative to the dose of each drug alone (Table 7). It is, however, not possible to determine the proportions of the mechanistic contributions of each drug that leads to the observed synergism or antagonism [16]. Furthermore, the DRI is not necessarily an indicator of synergism as an additive or slight antagonistic effect can also yield a DRI greater than 1. The DRI is therefore most relevant to in vivo experiments and would indicate that the dose of the drug used would reduce the chances for toxicity toward the host. Ideally, a combination of two drugs should yield strong synergism with a DRI > 1, a CI < 1 toward cancer cells, and a CI > 1 toward host cells [16]. From the results of this study, most of the cisplatin: BC-7 combinations indicated synergism (CI < 1) at an inhibition of 10%, 25%, 50%, and 75% of HeLa cell growth (Table 7). This synergism was also accompanied by a great dose reduction, especially in the dose of cisplatin.

## 4. Materials and Methods

### 4.1. Reagents

All cell lines mentioned below were purchased from Highveld Biological, Johannesburg, South Africa (Vero and HepG2), Cellonex, Johannesburg, South Africa (HeLa), or Japanese Collection of Research Bioresources Cell Bank (MeWo and MRHF), Sekisui XenoTech, LLC., West Cambridge Circle Dr. Kansas City, KS, USA). RPMI 1640, EMEM, DMEM low glucose cell culture medium, MEM NEAA, and FBS was purchased from GE Healthcare Life Sciences (Logan, UT, USA). Trypsin-EDTA, Dulbecco’s phosphate buffered saline (DPBS) with Ca^2+^ and Mg^2+^ and DPBS without Ca^2+^ and Mg^2+^ were purchased from Lonza (Wakersville, MD, USA). BisBenzamide H 33342 trihydrochloride (Hoechst 33342), cisplatin, penicillin/streptomycin, chloroquine, mangiferin, curcumin, carbonyl cyanide 3-chlorophenylhydrazone (CCCP), and bovine serum albumin fraction V (BSA) were purchased from Sigma-Aldrich (St. Louis, MO, USA). NucRed™ Live 647, CellRox^®^ Orange reagent, Lysotracker™ Deep Red, and Tetramethylrhodamine ethyl ester (TMRE) were purchased from Molecular Probes^®^-Life Technologies-Thermo Fisher Scientific (Logan, UT, USA). Annexin V-FITC/PI kit was purchased from MACS Miltenyi Biotec (Cologne, Germany). Cleaved caspase 3 (Asp175) (D3E9) Rabbit mAb, Cleaved caspase 8 (Asp391) (18C8) Rabbit mAb, Phospho-NF-κB p65 (Ser536) Rabbit mAb, Anti-rabbit IgG (H + L), F(ab’)2 fragment (Alexa fluor^®^ 647 conjugate), Anti-rabbit IgG (H + L), F(ab’)2 fragment (Alexa fluor^®^ 488 conjugate), and Phospho-Histone H3 (Ser10) Rabbit mAb were purchased from Cell Signalling Technology (Danvers, MA, USA).

### 4.2. Compound Preparation

BC-7 is a synthetic pyrazolyl thiourea derivative that has been synthesised through rapid ultrasound mediated methods as described by Nitulescu et al. [12].

### 4.3. Cell Culture Conditions

Cells were maintained in 10 cm culture dishes in complete DMEM low glucose (Vero, MRHF and MeWo), EMEM (HepG2), or RPMI 1640 (HeLa) supplemented with 10% foetal bovine serum (FBS) (Biowest) and incubated at 37 °C in a humidified incubator with 5% CO_2_. Additional supplementation, in the form of MEM Non-Essential Amino Acids at 1X with the stock concentration (HyClone), was required for HepG2 cells [34]. Cells exceeding 80% confluency were never used for maintenance and seeding of 96-well plates. Transfer numbers ranged from 138–148. Cells were investigated for mycoplasma using the Hoechst 33342 nuclear dye at a magnification of 60×. A positive test would indicate extra-nuclear fluorescence of mycoplasma DNA, either small cocci or filaments, whereas a negative test would indicate fluorescing nuclei against a dark background.

### 4.4. Experimental Imaging and Analysis

The ImageXpress Micro XLS Widefield High-Content Analysis System (Molecular Devices^®^, San Jose, CA, USA) together with the MetaXpress^®^ High-Content Image Acquisition and Analysis Software was used to acquire and analyse all images, for all experiments [34]. All assays were conducted in 96-well plates of which the area of one well was approximately 34.21 mm^2^. During image acquisition, 9 sites per well were imaged with a combined area of 21.72 mm^2^. Therefore, approximately 63.49% of the total area of the well was imaged. It is important to note that the number of cells following a 24 and/or 48-h treatment period after seeding at a specific density, as mentioned for each assay, is dependent on the doubling time of each cell line and the cytostatic or cytotoxic effect of a treatment, respectively.

### 4.5. Cytotoxicity

The cytotoxic activities of cisplatin and BC-7 on HeLa, MeWo, HepG2, Vero, and MRHF cells were assessed by the Hoechst 33342/PI method. Cells were seeded in 96-well plates at densities of 5000 cells/well (HeLa, Vero and MRHF) and 10000 cells/well (MeWo and HepG2) using 100 μL of aliquots and left overnight to attach. Treatment concentrations ranged from 0.1–200 μM for both cisplatin and BC-7. After 48 h, the treatment medium was aspirated and replaced with 100 μL of DPBS with Ca^2+^ and Mg^2+^ containing Hoechst 33342 (5 μg/mL) and incubated for 30 min at 37 °C. Prior to image acquisition, 10 μL aliquots of a 110 μg/mL PI stock was added to yield a final concentration of 10 μg/mL.

### 4.6. Selectivity Index

The selectivity index was calculated in order to determine the cytotoxic selectivity of BC-7 for cancer cells relative to normal cells according to the following equation:

SI = IC_50_^normal cells^/IC_50_^cancer cells^ (1)

The above equation was adapted to determine the selectivity of BC-7 for HeLa cells relative to MeWo and HepG2 cells:

SI = IC_50_^cancer cells (X)^/IC_50_^cancer cells (HeLa)^ (2)

where X refers to either MeWo or HepG2 cells and a SI < 2 was considered as general toxicity according to Suffness and Pezutto [14].

### 4.7. Mechanism of Cell Death Induction

#### 4.7.1. Cell Seeding and Treatment

HeLa cells were used for all subsequent assays and were seeded as described for cytotoxicity. Cells were treated with the respective IC_10_, IC_25_, IC_50_, and IC_75_ concentrations of BC-7 or cisplatin for 24 and 48 h.

#### 4.7.2. Cell Cycle Analysis and Micronuclei Formation

The NucRed™ Live 647 staining method was used to assess cell cycle arrest and micronuclei formation (as per manufacturer’s instructions). Following treatment, the cells were fixed with 4% formaldehyde for 15 min at room temperature. Cells were stained using 100 μL of DPBS with Ca^2+^ and Mg^2+^ containing NucRed™ Live 647 (50 μL per 10 mL) and incubated for 15 min at room temperature prior to imaging.

#### 4.7.3. Histone H3 Phosphorylation

Histone H3 phosphorylation was detected using the Phospho-Histone H3 (Ser10) Rabbit mAb (Cell Signalling Technology). Cells were fixed and stained with NucRed^™^ Live 647 as described for cell cycle analysis. After NucRed^™^ Live 647 staining, the cells were permeabilised using 80% ice cold methanol at −20 °C for 10 min. Cells were blocked using DPBS with Ca^2+^ and Mg^2+^ containing 0.5% BSA and thereafter incubated with primary antibody (1:250) for 1 h at 37 °C. A conjugated secondary antibody (1:1000), anti-rabbit IgG (H + L), F(ab’)2 Fragment (Alexa Fluor^®^ 488 Conjugate) was added and cells were incubated for 30 min at 37 °C in the dark. Prior to imaging, cells were washed twice with DPBS containing Ca^2+^ and Mg^2+^ to eliminate unbound antibody.

#### 4.7.4. Phosphatidylserine Translocation

Phosphatidylserine translocation (PS) was detected after 24 and 48 h using the Annexin V-FITC/PI kit from MACS Miltenyi Biotec with modifications. Treatment medium was removed, and cells were stained using a mixture of Annexin V-FITC (final concentration of 1× the stock) and Hoechst 33342 (final concentration of 2 μg/mL) for 15 min at room temperature. PI (final concentration of 1 μg/mL) was added to the Annexin/Hoechst 33342 stain in the 96-well plate just before image acquisition.

#### 4.7.5. Mitochondrial Membrane Potential Analysis

Mitochondrial membrane depolarisation was measured with TMRE. Staining was performed using 0.25 μM of TMRE and 2 μg/mL of Hoechst 33342 for 30 min at 37 °C prior to imaging. CCCP (1.56 μM) was included as a positive control for mitochondrial membrane depolarisation. Cells were exposed to CCCP for 1 h prior to staining.

#### 4.7.6. Caspase 8 and 3 Activation

Fixation, permeabilization, blocking, and staining were performed as described for Histone H3 phosphorylation. Cleaved caspase 8 (Asp391) (1:100) and cleaved caspase 3 (Asp175) (1:200) rabbit monoclonal antibodies (Cell Signalling Technology) were used for staining, respectively. Cells were incubated with either caspase 8 or 3 primary antibody for 1 h at 37 °C after which a conjugated secondary antibody (1:500), anti-rabbit IgG (H + L), F(ab’)2 Fragment (Alexa Fluor^®^ 647 Conjugate) was added for 30 min at 37 °C. Hoechst 33342 was used as a counterstain at a final concentration of 2 μg/mL prior to image acquisition.

#### 4.7.7. Reactive Oxygen Species Production

Staining was performed using 2.5 μM of CellRox^®^ Orange and 2 μg/mL of Hoechst 33342 for 30 min at 37 °C prior to imaging.

#### 4.7.8. Induction of Autophagy-Related Processes

Cells were stained using 50 nM of LysoTracker^™^ Deep Red and 2 μg/mL of Hoechst 33342 for 30 min at 37 °C prior to image acquisition. Chloroquine (100 μM) was included as a positive control for increasing the number of acidic organelles.

#### 4.7.9. Nuclear Factor kappa B Activation

After 24 and 48 h, cells were fixed, permeabilised, and blocked as described for Histone H3 phosphorylation. Cells were incubated with phospho-NF-κB p65 (Ser536) Rabbit mAb (1:800) for 1 h at 37 °C. Cells were washed and incubated with a conjugated secondary antibody (1:1000), anti-rabbit IgG (H + L), F(ab’)2 Fragment (Alexa Fluor^®^ 488 Conjugate) for 30 min at 37 °C in the dark. After staining with the secondary antibody, the cells were washed twice to eliminate unbound antibody and Hoechst 33342 (2 μg/mL) was used as a counterstain prior to imaging. Mangiferin (50 μM) was included as positive control for inhibition of nuclear factor kappa B (NF-κB).

### 4.8. Combination Treatment

HeLa cells were seeded in 96-well plates at a density of 5000 cells/well using 100 μL aliquots and left overnight to attach. For treatment, an additional 100 μL of the combination treatments were added. A total of 5 combination mixtures were made using the respective IC_50_ values of each compound at ratios of 1:3, 1:2, 1:1, 2:1, and 3:1, respectively. A ratio of 1:1 refers to IC_50_:IC_50_ whereas 1:3 means IC_50_:3 × IC_50_. These combination mixtures were serially diluted until 7 concentrations were achieved. Cells were incubated at 37 °C in a humidified 5% CO_2_ incubator for 48 h. Treatment medium was replaced with 100 μL of DPBS with Ca^2+^ and Mg^2+^ containing Hoechst 33342 at a final concentration of 5 μg/mL. PI was added to a final concentration of 10 μg/mL using 10 μL of per well of a 110 μg/mL stock prior to image acquisition.

#### 4.8.1. Determination of Combination Index

The term combination index (CI) was introduced as a quantitative method for determining synergism or antagonism between two drugs [35]. The CI theorem was derived by merging the principle of the mass-action law with the mathematical principle of induction and deduction. The derived general theory of dose and effect was also proven to be a unified theory of the four basic equations pioneered by Henderson-Hasselbalch, Michaelis-Menten, Hill, and Scatchard. The equation for the CI theorem is:

CI = [(*D*)_1_/(*D*x)_1_ + (*D*)_2_/(*D*x)_2_] (3)

where (*D*)_1_ and (*D*)_2_ denotes the dose of each drug in combination necessary to produce the same effect, and (*D*x)1 and (*D*x)2 denotes the individual dose of each drug required to inhibit a given level of cell growth and is determined by:

*Dx* = *D_m_*[*f*a/(1 − *f*a)]^1/*m*^ (4)

where *D_m_* refers to the potency (IC_50_), *m* to the shape, and *f*a denotes the fraction affected in the dose-effect curves.

The combined effect can then be indicated as CI < 1, synergism; CI = 1, additive effect, or CI > 1, antagonism, respectively.

#### 4.8.2. Determination of Dose Reduction Index

The dose reduction index (DRI) is defined as the level of dose reduction that is possible in a combination for a given level of effect as compared to the concentration of the individual drug alone. The equation for the DRI is:

(*DRI*)_1_ = (*Dx*)_1_/(*D*)_1_ (5)

(*DRI*)_2_ = (*Dx*)_2_/(*D*)_2_ (6)

A value of DRI > 1 is considered favourable [36].

### 4.9. Statistical Analysis

All experiments were performed at least 3 times in which 3 different transfer numbers of HeLa cells were used. Statistical analysis was performed by means of One-way Anova in conjunction with the Dunnet post-test and confirmed with the two-tailed student t-test for two samples assuming equal variance. Error bars represent the standard deviation of the mean (SD, *n* = 3). IC_50_ values were calculated from 6-point log-dose response curves using GraphPad Prism version 5.01. IC_10_, IC_25_, and IC_75_ values were calculated using the GraphPad QuickCalcs: Compute ECanything from EC_50_ calculator available at http://www.graphpad.com/quickcalcs/Ecanything1/. IC_10_, IC_25_, IC_50_, and IC_75_ values for combination treatments were manually calculated from median-effect plots generated from 7-point log-dose response curves for each combination ratio.

## 5. Conclusions

In conclusion, this was the first time that BC-7 was evaluated for its ability to induce Histone H3 phosphorylation, micronuclei formation, mitochondrial membrane depolarisation, autophagy induction, ROS production, and NF-κB activation. It was shown that BC-7 induced apoptosis in a mitochondrial- and caspase-dependent manner possibly preceded by the onset of mitotic catastrophe as supported by the formation of micronuclei. Treatment with BC-7 was also shown to induce autophagy and the activation of NF-κB. Cell cycle arrest occurred in the early M phase after 24 and 48 h with a noticeable increase in G2 arrest after 48 h.

Combination treatments also indicated, for the first time, that BC-7 may have the ability to function in a synergic manner with cisplatin and suggests that it could lower the dose of cisplatin in combination to achieve a similar anti-cancer efficacy compared to the higher cisplatin dose when used alone. The lower dosage in combination could result in reduced drug resistance as well as limit the toxicity on normal cells (yet to be confirmed) associated with cisplatin treatment.

## Figures and Tables

**Table 1:** IC_10_, IC_25_, IC_50_, and IC_75_ values for 5-aminopyrazole derivative *N*-[[3-(4-bromophenyl)-1*H*-pyrazol-5-yl]-carbamothioyl]-4-chloro-benzamide (BC-7) and cisplatin as determined from dose response curves for HeLa cells.

Treatment	IC_10_	IC_25_	IC_50_	IC_75_
Cisplatin (μM)	0.069 ± 0.012	0.342 ± 0.060	1.675 ± 0.301	8.194 ± 1.499
BC-7 (μM)	18.50 ± 3.520	34.83 ± 5.437	65.58 ± 8.400	123.5 ± 12.98

**Table 2:** SI (selectivity index) values for BC-7 and cisplatin for HeLa cells compared to MeWo, HepG2, Vero, and MRHF cells.

Treatment	Vero	MRHF	MeWo	HepG2
Cisplatin (μM)	3.53	3.87	5.55	2.10
BC-7 (μM)	2.58	1.70	>3.05	>3.05

**Table 3:** Changes in phosphorylated Histone H3 levels in HeLa cells after 24 and 48 h of exposure to BC-7 and cisplatin.

Treatment	Cells Stained Positive for Phosphorylated Histone H3 (%)			
	Cisplatin		BC-7	
	24 h	48 h	24 h	48 h
control	12.28 ± 1.88	5.35 ± 0.52	12.28 ± 1.88	5.35 ± 0.52
IC_10_	13.01 ± 1.35	5.32 ± 0.99	16.57 ± 0.58 *	5.83 ± 0.44
IC_25_	11.95 ± 0.76	5.83 ± 1.23	16.71 ± 0.61 *	5.74 ± 0.23
IC_50_	7.90 ± 0.52 *	8.70 ± 1.42 *	17.40 ± 0.77 *	5.39 ± 0.28
IC_75_	6.04 ± 1.16 *	4.55 ± 1.47	15.42 ± 0.47 *	5.89 ± 0.24

Control treatment refers to untreated control. Significance was determined using the two-tailed student *t*-test: * *p* < 0.05.

**Table 4:** Changes in mitochondrial membrane potential (MMP) in HeLa cells after 24 and 48 h of exposure to cisplatin and BC-7.

Treatment	Tetramethylrhodamine Ethyl Ester (TMRE) Mean Cytoplasmic Integrated Intensity (% of Control)			
	Cisplatin		BC-7	
	24 h	48 h	24 h	48 h
control	100 ± 9.49	100 ± 3.29	100 ± 9.49	100 ± 3.29
IC_10_	72.17 ± 6.75 *	86.62 ± 2.04 **	94.85 ± 1.74	64.55 ± 1.72 **
IC_25_	81.95 ± 5.54 *	88.00 ± 1.04 **	92.28 ± 1.83	63.83 ± 3.45 **
IC_50_	80.65 ± 6.28 *	76.30 ± 1.85 **	90.74 ± 2.30	61.63 ± 0.06 **
IC_75_	77.33 ± 2.98 *	62.87 ± 0.04 **	77.60 ± 2.05 *	42.39 ± 3.33 **

Control treatment refers to untreated control. Significance was determined using the two-tailed student *t*-test: * *p* < 0.05 and ** *p* < 0.005 compared to untreated control.

**Table 5:** Changes in cleaved caspase 8 levels in HeLa cells after 24 and 48 h of exposure to cisplatin and BC-7.

Treatment	Cleaved Caspase 8 Mean Cell Integrated Intensity (% of Control)			
	Cisplatin		BC-7	
	24 h	48 h	24 h	48 h
control	100 ± 1.15	100 ± 1.82	100 ± 1.15	100 ± 1.82
IC_10_	99.14 ± 1.23	102.9 ± 1.46	108.1 ± 8.25	113.3 ± 1.77 **
IC_25_	105.9 ± 2.66 *	108.2 ± 2.77 *	111.6 ± 6.46 *	118.2 ± 6.32 **
IC_50_	116.3 ± 0.21 **	151.8 ± 8.08 **	113.4 ± 10.8	117.0 ± 3.00 **
IC_75_	124.7 ± 2.24 **	167.2 ± 7.37 **	114.0 ± 9.81	119.3 ± 0.96 **

Control treatment refers to untreated control. Significance was determined using the two-tailed student *t*-test: * *p* < 0.05 and ** *p* < 0.005 compared to untreated control.

**Table 6:** Changes in cleaved caspase 3 levels in HeLa cells after 24 and 48 h of exposure to cisplatin and BC-7.

Treatment	Cleaved Caspase 3 Mean Cell Integrated Intensity (% of Control)			
	Cisplatin		BC-7	
	24 h	48 h	24 h	48 h
control	100 ± 4.86	100 ± 1.17	100 ± 4.86	100 ± 1.17
IC_10_	103.5 ± 5.20	105.1 ± 1.94 *	103.4 ± 5.43	107.7 ± 1.83 **
IC_25_	110.6 ± 5.94	111.2 ± 3.29 *	104.8 ± 5.02	115.7 ± 1.62 **
IC_50_	123.2 ± 5.28 *	155.7 ± 7.82 **	103.7 ± 4.75	116.4 ± 2.94 **
IC_75_	129.1 ± 6.23 **	174.0 ± 8.58 **	110.4 ± 5.72	117.6 ± 0.45 **

Control treatment refers to untreated control. Significance was determined using the two-tailed student *t*-test: * *p* < 0.05 and ** *p* < 0.005 compared to untreated control.

**Table 7:** Combination index (CI) and dose reduction index (DRI) values for combinations of cisplatin: BC-7 at inhibition of 10%, 25%, 50%, and 75% of HeLa cell growth.

Ratio (Cisplatin:BC-7)	CI ^a^ Values at Inhibition of				DRI ^b^ Values at Inhibition of			
	10%	25%	50%	75%	10%	25%	50%	75%
1:3	0.21	0.32	0.61	1.38	6.90:15.7	6.76:5.86	6.56:2.19	6.37:0.82
1:2	0.27	0.46	0.96	2.45	4.73:16.1	3.86:5.02	3.13:1.56	2.53:0.49
1:1	0.71	0.58	0.58	0.72	1.62:11.1	2.38:6.20	3.46:3.46	5.01:1.92
2:1	0.54	0.51	0.54	0.70	1.97:27.0	2.36:12.3	2.79:5.59	3.30:2.54
3:1	0.78	0.61	0.53	0.55	1.35:27.7	1.85:14.4	2.51:7.52	3.39:3.92

^a^ CI < 0.3: Strong synergism; CI < 0.7: Synergism; CI < 0.9: Moderate/Slight synergism. ^b^ DRI < 1: Unfavourable.

## Supplementary Materials

The following are available online at https://www.mdpi.com/1422-0067/20/22/5559/s1, Figure S1: Cell cycle analysis of HeLa cells after 48 h treatments with BC-7 and cisplatin, Figure S2: NF-κB analysis in HeLa cells after 24 and 48 h of treatment with curcumin and mangiferin, Figure S3: Micrographs (10× magnification) indicating positive staining for acidic organelles in HeLa cells after 48 h of treatment compared to control. Figure S4: Average number of HeLa cells per site after 48 h of treatment with cisplatin: BC-7 combinations at different ratios, Figure S5: Nuclei mean area of HeLa cells after 48 h of treatment with cisplatin: BC-7 combinations at different ratios.
