# Supplementary material for: Anti-Cancer Activity of a 5-Aminopyrazole Derivative Lead Compound (BC-7) and Potential Synergistic Cytotoxicity with Cisplatin against Human Cervical Cancer Cells
Source: Int J Mol Sci. 2019 Nov 7;20(22):5559. doi: 10.3390/ijms20225559 (PMC6888365; doi:10.3390/ijms20225559)
Supplement: Supplementary file 1 [file ijms-20-05559-s001.pdf]

International Journal of
Molecular Sciences

MDPI

Supplementary Material

Article

Anti-Cancer Activity of a 5-Aminopyrazole Derivative Lead Compound (BC-7) and Potential Synergistic Cytotoxicity with Cisplatin against Human Cervical Cancer Cells

Bresler Swanepoel [1], George Mihai Nitulescu [2,*], Octavian Tudorel Olaru [2], Luanne Venables [1] and Maryna van de Venter [1]

[1] Department of Biochemistry and Microbiology, PO Box 77000, Nelson Mandela University, Port Elizabeth, 6031, South Africa; s211129399@mandela.ac.za (BS); s204004039@mandela.ac.za (L.V.); Maryna.VanDeVenter@mandela.ac.za (M.v.d.V.)

[2] Faculty of Pharmacy, "Carol Davila" University of Medicine and Pharmacy, Traian Vuia 6, Bucharest 020956, Romania; george.nitulescu@umfcd.ro (GMN); octavian.olaru@umfcd.ro (O.T.O.)

* Correspondence: george.nitulescu@umfcd.ro; nitulescu_mihai@yahoo.com

Supplementary Figures

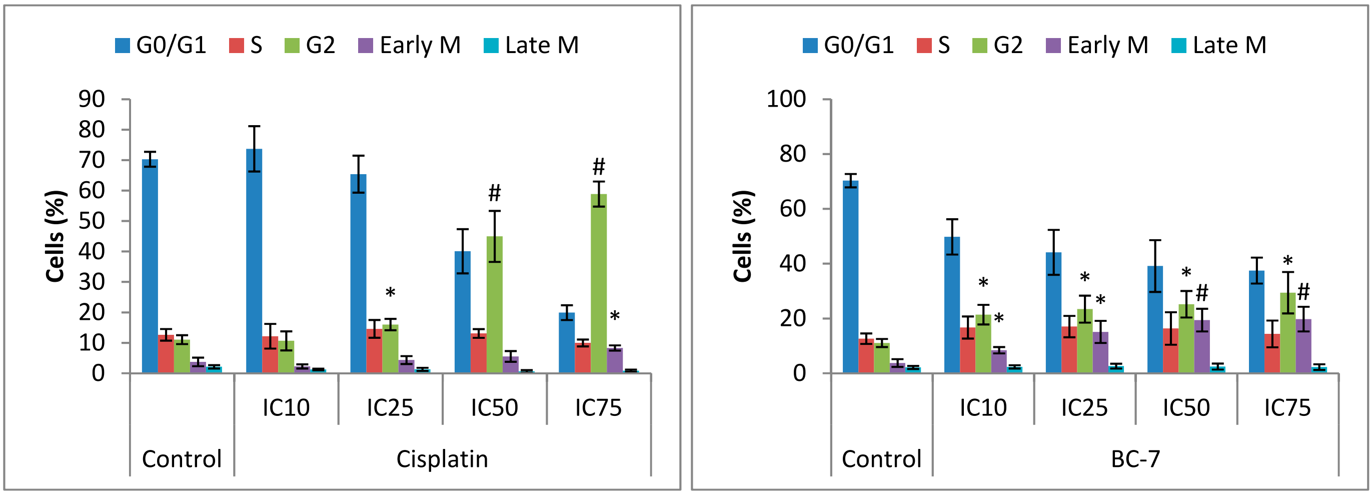

Figure S1. Cell Cycle Analysis of HeLa cells after 48 hour treatments with BC-7 and cisplatin. Cell cycle analysis was determined by the NucRed™ Live 647 staining method. Control treatment refers to untreated control. Results displayed as percentage of cells detected in each phase Error bars indicate SD of three individual experiments, each performed in quadruplicate ($n = 3$). Significance was determined using the two-tailed Student *t*-test: * $p < 0.05$ and # $p < 0.005$ compared to untreated control.

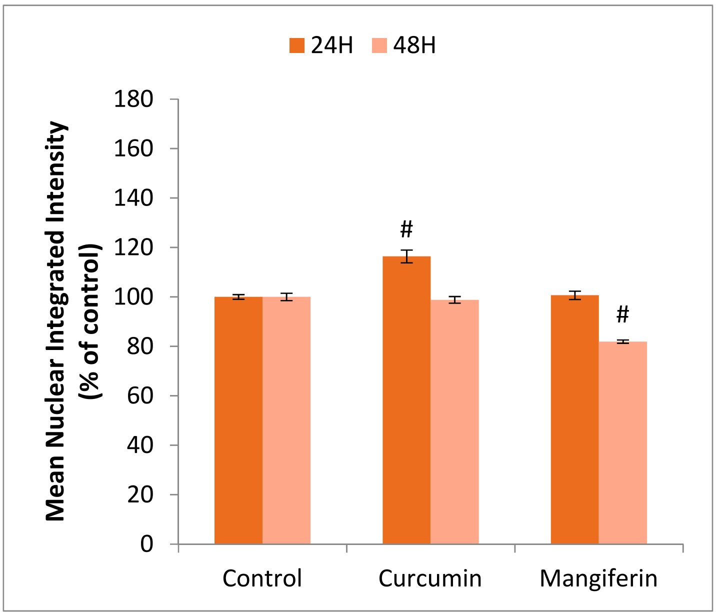

Figure S2. NF-kB analysis in HeLa cells after 24 and 48 hours of treatment with curcumin and mangiferin. Immunofluorescence staining with phospho-p65 NF-kB was done. Control treatment refers to untreated control. Results displayed as mean nuclear integrated intensity and expressed as a percentage of the untreated control. Error bars indicate SD of three individual experiments, each performed in quadruplicate (n = 3)**.** Significance was determined using the two-tailed Student t-test: * $p < 0.05$ and # $p < 0.005$ compared to untreated control.

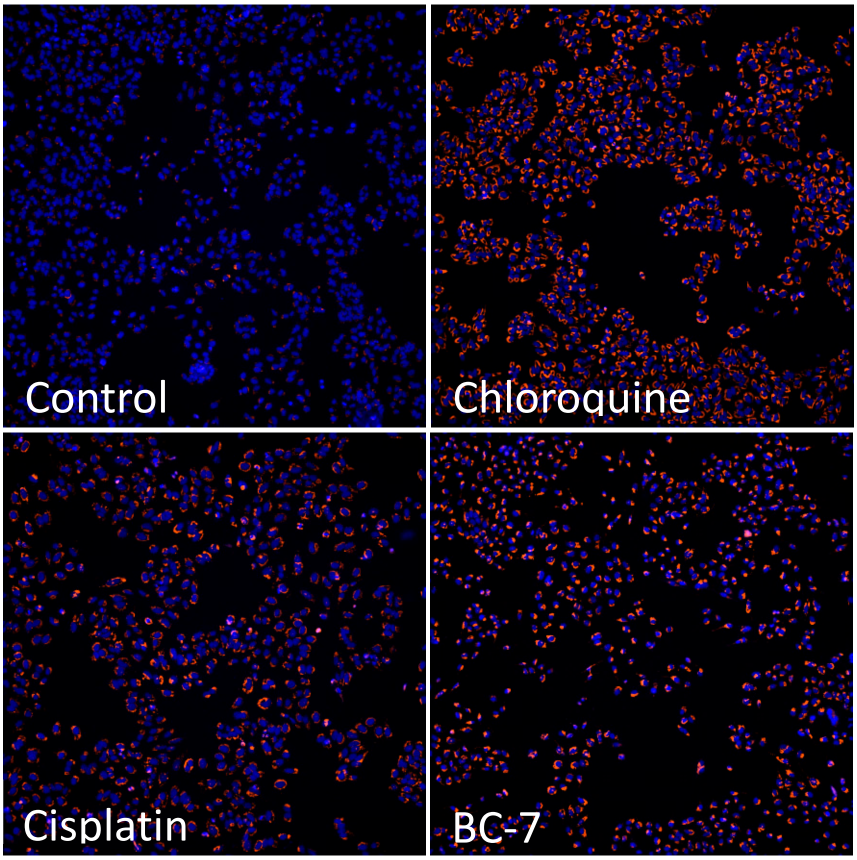

Figure S3. Micrographs (10x magnification) indicating positive staining for increased lysosomal content in HeLa cells after 48 hours of treatment compared to control. Control treatment refers to untreated control. Cells were stained with LysoTracker™ Deep Red and Hoechst 33342. Nuclei: Blue; acidic organelles: Orange.

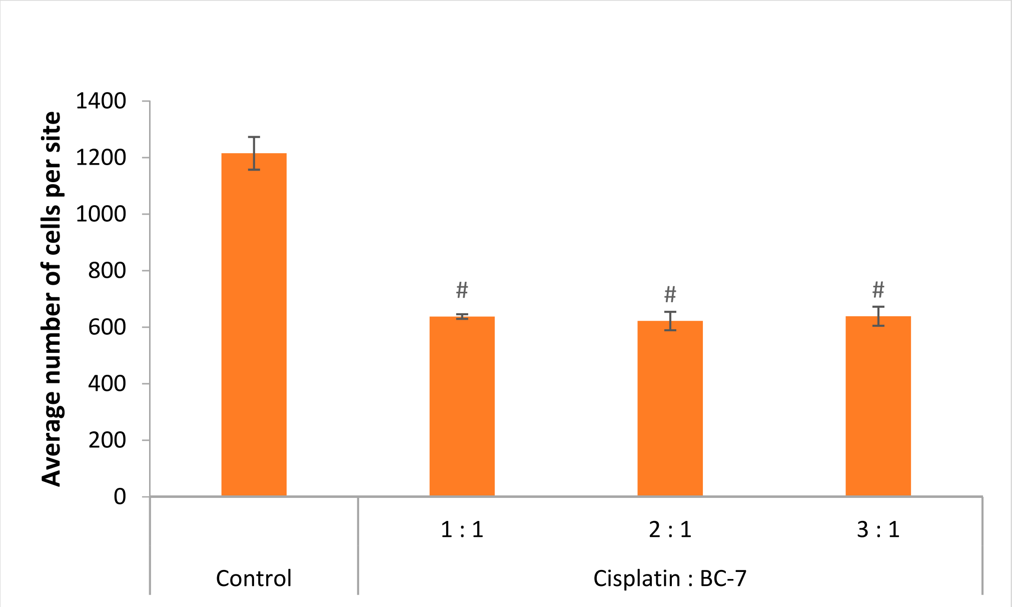

Figure S4. Average number of HeLa cells per site after 48 hours of treatment with cisplatin:BC-7 combinations at different ratios. Results expressed as average number of cells per site of 9 sites acquired per well of a 96-well plate. Control treatment refers to untreated control. Cells were stained with Hoechst 33342. Error bars indicate SD of three individual experiments, each performed in quadruplicate (n = 3). Significance was determined using the two-tailed Student t-test: * p <0.05 and # p < 0.005 compared to untreated control.

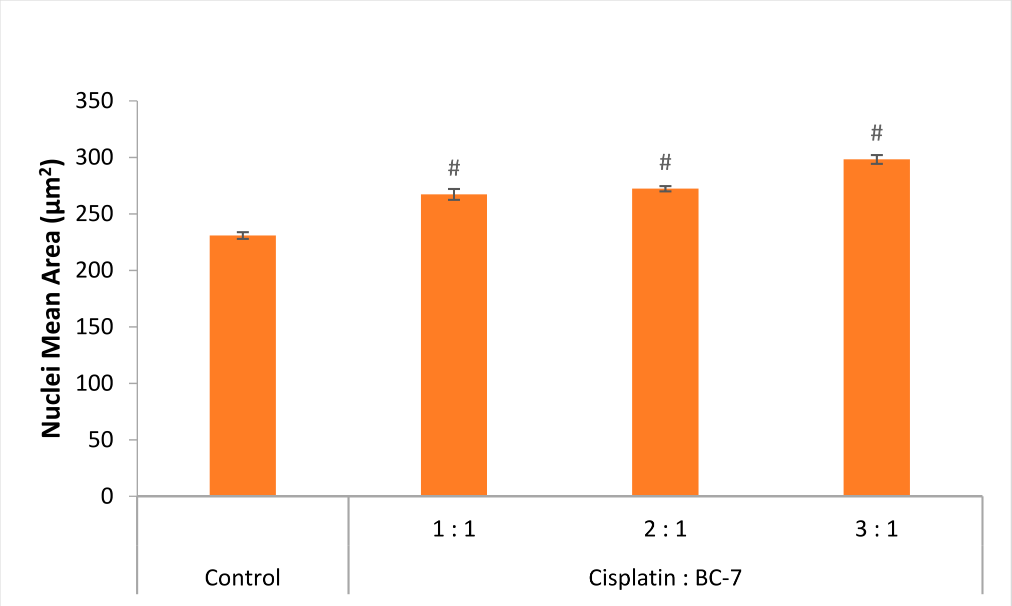

Figure S5. Nuclei mean area of HeLa cells after 48 hours of treatment with cisplatin:BC-7 combinations at different ratios. Control treatment refers to untreated control. Cells were stained with Hoechst 33342. Error bars indicate SD of three individual experiments, each performed in quadruplicate (n = 3). Significance was determined using the two-tailed Student t-test: * p < 0.05 and # p < 0.005 compared to untreated control.

© 2019 by the authors. Submitted for possible open access publication under the terms and conditions of the Creative Commons Attribution (CC BY) license (http://creativecommons.org/licenses/by/4.0/).
